# Anti-inflammatory Effects of Empagliflozin and Gemigliptin on LPS-Stimulated Macrophage via the IKK/NF-*κ*B, MKK7/JNK, and JAK2/STAT1 Signalling Pathways

**DOI:** 10.1155/2021/9944880

**Published:** 2021-06-02

**Authors:** Nami Lee, Yu Jung Heo, Sung-E Choi, Ja Young Jeon, Seung Jin Han, Dae Jung Kim, Yup Kang, Kwan Woo Lee, Hae Jin Kim

**Affiliations:** ^1^Department of Endocrinology and Metabolism, Ajou University School of Medicine, Suwon, Republic of Korea; ^2^Department of Physiology, Ajou University School of medicine, Suwon, Republic of Korea

## Abstract

**Background:**

Sodium-glucose cotransporter 2 (SGLT2) and dipeptidyl peptidase-4 (DPP-4) inhibitors are glucose-lowering drugs whose anti-inflammatory properties have recently become useful in tackling metabolic syndromes in chronic inflammatory diseases, including diabetes and obesity. We investigated whether empagliflozin (SGLT2 inhibitor) and gemigliptin (DPP-4 inhibitor) improve inflammatory responses in macrophages, identified signalling pathways responsible for these effects, and studied whether the effects can be augmented with dual empagliflozin and gemigliptin therapy.

**Methods:**

RAW 264.7 macrophages were first stimulated with lipopolysaccharide (LPS), then cotreated with empagliflozin, gemigliptin, or empagliflozin plus gemigliptin. We conducted quantitative RT-PCR (qRT-PCR) to determine the most effective anti-inflammatory doses without cytotoxicity. We performed ELISA and qRT-PCR for inflammatory cytokines and chemokines and flow cytometry for CD80, the M1 macrophage surface marker, to evaluate the anti-inflammatory effects of empagliflozin and gemigliptin. NF-*κ*B, MAPK, and JAK2/STAT signalling pathways were examined via Western blotting to elucidate the molecular mechanisms of anti-inflammation.

**Results:**

LPS-stimulated CD80^+^ M1 macrophages were suppressed by coincubation with empagliflozin, gemigliptin, and empagliflozin plus gemigliptin, respectively. Empagliflozin and gemigliptin (individually and combined) inhibited prostaglandin E_2_ (PGE_2_) release and COX-2, iNOS gene expression in LPS-stimulated RAW 264.7 macrophages. These three treatments also attenuated the secretion and mRNA expression of proinflammatory cytokines, such as TNF-*α*, IL-1*β*, IL-6, and IFN-*γ*, and proinflammatory chemokines, such as CCL3, CCL4, CCL5, and CXCL10. All of them blocked NF-*κ*B, JNK, and STAT1/3 phosphorylation through IKK*α*/*β*, MKK4/7, and JAK2 signalling.

**Conclusions:**

Our study demonstrated the anti-inflammatory effects of empagliflozin and gemigliptin via IKK/NF-*κ*B, MKK7/JNK, and JAK2/STAT1 pathway downregulation in macrophages. In all cases, combined empagliflozin and gemigliptin treatment showed greater anti-inflammatory properties.

## 1. Introduction

Certain aspects of chronic low-grade inflammation in obesity and metabolic syndrome-related diseases, such as hypertension, atherosclerosis, and diabetes mellitus, have been studied [1]. Some antidiabetic drugs exert anti-inflammatory properties that can be mediated by directly or indirectly regulating the inflammatory response, and recent studies show that sodium-glucose cotransporter 2 (SGLT2) and dipeptidyl peptidase-4 (DPP-4) inhibitors may exert potential anti-inflammatory functions [2–4].

Sodium-glucose cotransporters are a family of active glucose transporter proteins expressed in bacteria and animals, including 12 human genes [5, 6]. SGLT1 is expressed in numerous organs, including the small intestine, kidney, brain, heart, and immune cells [7–11], while SGLT2 is predominantly expressed in the kidney [12]. SGLT2 inhibitors were initially known to improve hyperglycemia through glycosuria by competitively inhibiting both SGLT1 and SGLT2 [13]. These antidiabetic agents have been recently reported to promote M2 macrophage polarization with an anti-inflammatory phenotype and to attenuate the production of proinflammatory cytokines on diabetic cardiomyopathy and nephropathy in mice [14, 15]. Two recent studies have shown evidence of SGLT2 protein expression in RAW 264.7 (mouse macrophage cell line) [16] and Kupffer (hepatic stellate macrophage) cells [17].

DPP-4, which exists both as a type II cell surface protein (CD26) and a soluble molecule lacking intracellular and membrane-anchoring domains, is ubiquitously expressed by numerous cells in rats, mice, and humans [18–24]. It acts as an enzymatic and nonenzymatic multifunctional protein, depending on the expressing cell type and cellular conditions, by regulating peptides or influencing cell signals [18, 25, 26]. DPP-4 aids macrophage and dendritic cell maturation, consequently inducing T-cell activation, and has recently emerged as an important inflammatory response regulator [24, 27, 28]. DPP-4 inhibitors, which have been initially approved as an antidiabetic drug, are now proposed as a nondiabetic drug for treatment of several inflammatory diseases [29–31]. DPP-4 inhibitors also attenuated NOD-like receptor pyrin domain-containing protein 3 (NLRP3) inflammasome activation, reducing proinflammatory cytokine productions in diabetic mouse kidney and heart [32, 33].

Combination therapy of SGLT2 and DPP-4 inhibitors has a stronger glucose-lowering effect than either monotherapy in type 2 diabetes patients due to different mechanisms and complementary effects [34]. This treatment strategy is expected to provide additional anti-inflammatory effects to reduce disease progression and risk of complications. However, the molecular mechanisms of SGLT2 and DPP-4 inhibitors for inflammatory disease have not been fully verified. Furthermore, it is rare to investigate the anti-inflammatory effect after direct treatment of macrophages with a combination of these two agents. Therefore, we evaluated the impact of SGLT2 inhibition, DPP-4 inhibition, and simultaneous SGLT2 and DPP-4 inhibition on proinflammatory response in LPS-stimulated macrophages and their potential mechanism.

## 2. Materials and Methods

### 2.1. Cell Culture and Reagents

RAW 264.7 murine macrophage cells were obtained from the American Type Culture Collection (Manassas, VA, USA). The macrophages were cultured in high-glucose Dulbecco's Modified Eagle's medium (DMEM; Welgene Inc., Daegu, South Korea) supplemented with 10% (*v*/*v*) fetal bovine serum (FBS; Grand Island, NY, USA) and antibiotics (10 *μ*g/mL streptomycin, and 100 IU/mL penicillin) at 37°C and 5% CO_2_ in a humidified atmosphere of 95% air. These cells were cultured to ≥85% confluence before treatment with empagliflozin (AdooQ Bioscience, Irvine, CA), gemigliptin (LG Life Sciences Ltd., Seoul, South Korea), lipopolysaccharide (LPS) from *Escherichia coli* 055:B5 (Sigma-Aldrich, St. Louis, MO, USA), or dexamethasone (Sigma-Aldrich). Empagliflozin and gemigliptin were dissolved in dimethyl sulfoxide (DMSO; Sigma-Aldrich) and added to the cell culture at the desired concentrations. The final DMSO concentration did not exceed 0.1%, and all samples were incubated with the same amounts of DMSO. LPS and dexamethasone were dissolved in phosphate-buffered saline (PBS; Welgene Inc.), and all additives were used as cotreatments following a 2-h period of cell starvation.

### 2.2. Cell Viability Assay

A Cell Counting Kit-8 (CCK-8) assay (Dojindo Laboratories, Kumamoto, Japan) was used to measure the cytotoxicity of the regents on RAW 264.7 macrophages. After incubation at 37°C for 24 h in 96-well plates at a density of 5 × 10^4^ cells/well, the cells were treated with reagents at 37°C for 48 h. Then, the cells were washed, and 10 *μ*L CCK-8 solution was added to each well, followed by incubation at 37°C for 2 h. The absorbance at 450 nm was determined by a microplate absorbance reader (Bio-Rad, Hercules, CA, USA). Treated cell viability was assessed as a percentage of the absorbance values compared to the control (untreated) cells.

### 2.3. Flow Cytometry Analysis

RAW 264.7 macrophages were collected after treatment for 48 h, and the cells were washed in PBS/2% FCS (flow cytometry staining buffer). The Fc region was blocked by incubating with anti-FcRII/III monoclonal antibodies (mAbs, clone 2.4G2; Invitrogen, Carlsbad, CA) and 10% normal mouse serum for 15 min on ice. Then, the cells were stained for 45 min on ice with FITC anti-CD80 (B7-1; Invitrogen) and fixed in PBS/1% paraformaldehyde. Twenty thousand cells were analysed on a FACSAria III cell sorter (BD Biosciences, San Jose, CA, USA), and data were analysed using FlowJo Vx (TreeStar Inc., Ashland, OR, USA).

### 2.4. Western Blot Assay

The cells were lysed in radioimmunoprecipitation assay (RIPA) buffer (150 mM NaCl, 1% (*v*/*v*) NP-40, 0.5% (*w*/*v*) deoxycholate, 0.1% (*w*/*v*) sodium dodecyl sulphate (SDS), 50 mM Tris-HCl (pH 7.5), and a protease inhibitor (Pancreas extract, Pronase, Thermolysin, Chymotrypsin, Papain) cocktail; Roche Applied Science, Mannheim, Germany) and incubated for 20 min on ice. Total proteins were then extracted by differential centrifugation at 13,000×g for 10 min, and the protein concentrations in lysates were determined using a protein assay kit (Bio-Rad, Hercules, CA, USA). Subsequently, the cell lysates were mixed with equal volumes of 2× SDS sample buffer (125 mM Tris-HCl (pH 6.8), 4% (*w*/*v*) SDS, 4% (*v*/*v*) 2-mercaptoethanol, and 20% (*v*/*v*) glycerol). The cell lysates, containing equivalent amounts of 20 *μ*g protein, were subjected to 8–12% (*w*/*v*) sodium dodecyl sulphate-polyacrylamide gel electrophoresis (SDS-PAGE) and transferred to polyvinylidene fluoride (PVDF) membranes (Millipore, Billerica, MA, USA). The membranes were blocked in 5% (*w*/*v*) skim milk or bovine serum albumin (BSA) for 30 min and then incubated overnight with primary antibodies, against cyclooxygenase-2 (COX-2), inducible nitric oxide synthase (iNOS), phosphor-nuclear factor kappa-light-chain-enhancer of activated B cells (NF-*κ*B), total NF-*κ*B, phosphor- inhibitor of NF-*κ*B kinase (IKK)*α*/*β*, total IKK*α*, total IKK*β*, phosphor-c-Jun N-terminal kinase (JNK), total JNK, phosphor-p38 mitogen-activated protein kinase (p-38), total p-38, phosphor-mitogen-activated protein kinase kinase 4 (MKK4), total MKK4, phophor-MKK7, total MKK7, phosphor-signal transducer and activator of transcription 1 (STAT1), total STAT1, phosphor-STAT3, total STAT3, phosphor-janus family tyrosine kinase 2 (JAK2), total JAK2, glyceraldehyde 3-phosphate dehydrogenase (GAPDH) (Cell Signaling Technology, Danvers, MA, USA), phosphor-extracellular signal-regulated kinase (ERK), and total ERK (Santa Cruz Biotechnology, Santa Cruz, CA, USA) at 4°C. The membranes were then incubated with the secondary antibodies (horseradish peroxidase-conjugated anti-goat IgG and anti-rabbit IgG (Bethyl Laboratories, Montgomery, TX, USA)). Finally, the blots were measured and visualized using an enhanced chemiluminescence kit (Amersham Pharmacia Biotech, Piscataway, NJ, USA).

### 2.5. RNA Isolation and Quantitative Real-Time Reverse Transcriptase-Polymerase Chain Reaction (qRT-PCR) Analysis

The total RNAs were extracted using the RNAiso Plus reagent (Takara Bio, Otsu, Japan) according to the manufacturer's protocol. Complementary DNA (cDNA) was synthesized from RNA using avian myeloblastosis virus (AMV) reverse transcriptase (BEAMS Biotechnology, Seongnam, South Korea), and random 9-mer primers, then amplified qPCR using primer sets (Takara Bio Inc., Shiga, Japan) specific for mouse COX-2: CAG CAA ATC CTT GCT GTT CC (forward, F) and TGG GCA AAG AAT GCA AAC ATC (reverse, R); mouse iNOS: AGA CCT CAA CAG AGC CCT CA (F) and GGC TGG ACT TTT CAC TCT GC (R); mouse tumor necrosis factor-*α* (TNF-*α*): TCG TAG CAA ACC ACC AAG TG (F) and AGA TAG CAA ATC GGC TGA CG (R); mouse interleukin (IL)-1*β*: TCT CGC AGC AGC ACA TCA ACA (F) and CCT GGA AGG TCC ACG GGA AA (R); mouse IL-6: GAC CTG TCT ATA CCA CTT CAC (F) and GTG CAT CAT CGT TGT TCA TAC (R); mouse C-C motif chemokine ligands (CCL) 3: ACT GCC CTT GCT GTT CTT CT (F) and GTC TCT TTG GAG TCA GCG CA (R); mouse CCL4: CTC TCT CCT CTT GCT CGT GG (F) and CTC ACT GGG GTT AGC ACA GA (R); mouse CCL5: CCA TCA TCC TCA CTG CAG CC (F) and CTC TGG GTT GGC ACA CAC TT (R); and mouse C-X-C motif chemokine ligand (CXCL) 10: CCA AGT GCT GCC GTC ATT TT (F) and TCA TCA TTC TTT TTC ATC GTG GCA (R). Quantitative real-time PCR was performed using SYBR Green Master Mix (Takara Bio Inc., Tokyo, Japan) on a Takara TP-815 instrument. Relative expression levels were determined in comparison with the control, using GAPDH as an internal control.

### 2.6. Enzyme-Linked Immunosorbent Assay (ELISA)

The supernatants from differential centrifugation were used to determine the protein levels of mouse prostaglandin E_2_ (PGE_2_), TNF-*α*, IL-1*β*, IL-6, and interferon-*γ* (IFN-*γ*) using the DuoSet ELISA kit (R&D Systems Inc., Minneapolis, MN, USA) according to the manufacturer's instructions.

### 2.7. Statistical Analysis

All experiments were conducted in triplicates, and the data were presented as the means ± standard deviation. The results were analysed using Student's *t*-test, and results with *p* values of ≤0.05 were considered statistically significant.

## 3. Results

### 3.1. Effect of Empagliflozin and Gemigliptin on LPS-Stimulated Inflammation in RAW 264.7 Macrophages

**Figure 1:**
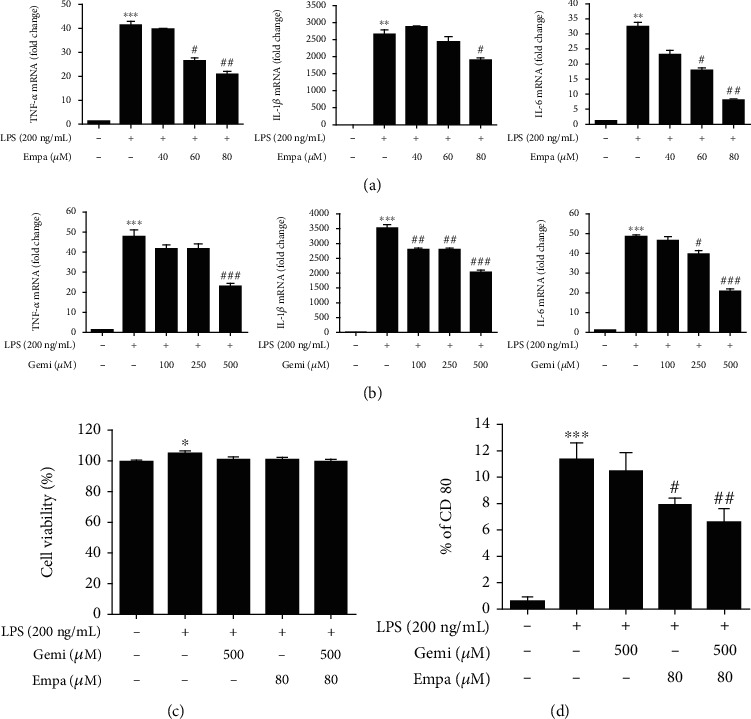
Empagliflozin and gemigliptin diminished LPS-induced proinflammatory response in RAW 264.7 macrophages. Cells were treated with LPS (200 ng/mL) alone, plus the indicated concentrations of (a) empagliflozin (Empa) or (b) gemigliptin (Gemi) for 4 h and proinflammatory cytokines, TNF-*α*, IL-1*β*, and IL-6; mRNA expression was analyzed using real-time PCR. (c) Macrophages were treated with LPS alone, plus 500 *μ*M Gemi, 80 *μ*M Empa, or both Gemi and Empa (Gemi+Empa) for 48 h. Cell viabilities were determined via CCK-8 assay. (d) M1 macrophage marker, CD80, was assessed via flow cytometry after 48 h treatment. Data are presented as the means ± standard errors from three independent experiments. ^∗^*p* < 0.05, ^∗∗^*p* < 0.01, and ^∗∗∗^*p* < 0.001 indicate comparison with the untreated control. ^#^*p* < 0.05, ^##^*p* < 0.01, and ^###^*p* < 0.001 indicate results with statistically significant differences between the LPS-stimulated control and Gemi-, Empa-, or Gemi+Empa-treated groups. ^†^*p* < 0.05, ^††^*p* < 0.01, and ^†††^*p* < 0.001 indicate results with statistically significant differences between Empa- and Gemi+Empa-treated groups.

To determine the effective concentration of empagliflozin and gemigliptin without cell toxicity, RAW 264.7 macrophages were treated with the various doses of empagliflozin (40, 60, and 80 *μ*M) or gemigliptin (100, 250, and 500 *μ*M) for 4 h (Figures 1(a) and 1(b)) [16, 35]. Treatment of LPS-stimulated RAW 264.7 macrophages with 80 *μ*M empagliflozin or 500 *μ*M gemigliptin significantly inhibited mRNA expression of proinflammatory cytokines, without affecting cell viability for 48 h (Figure 1(c)). Thus, these concentrations were used in subsequent experiments. We confirmed that empagliflozin and gemigliptin inhibited the proinflammatory response by reducing the number of M1 macrophage surface marker, CD80, expressing cells in LPS-induced RAW 264.7 macrophages after 48 h treatment (Figure 1(d)) [36].

### 3.2. Empagliflozin and Gemigliptin Inhibited PGE_2_ Production and Attenuated COX-2 and iNOS mRNA Expression in LPS-Activated RAW 264.7 Macrophages

**Figure 2:**
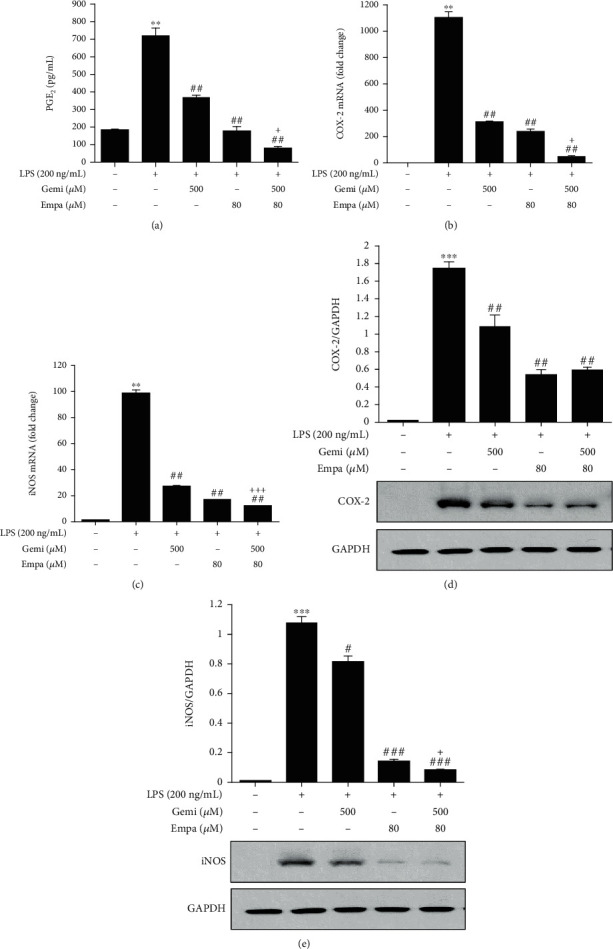
Empagliflozin and gemigliptin reduced PGE_2_ release and suppressed COX-2 and iNOS gene expression in LPS-activated RAW 264.7 macrophages. (a) Culture supernatants were collected after 48 h treatment, and PGE_2_ concentration was measured using ELISA. (b) COX-2 and (c) iNOS mRNA expression levels were analysed via real-time PCR after 4 h treatment. (d, e) Western blot assays were conducted to evaluate protein band quantity by densitometry. Data are presented as the means ± standard errors from three independent experiments. ^∗^*p* < 0.05, ^∗∗^*p* < 0.01, and ^∗∗∗^*p* < 0.001 indicate comparison with the untreated control. ^#^*p* < 0.05, ^##^*p* < 0.01, and ^###^*p* < 0.001 indicate results with statistically significant differences between the LPS-stimulated control and Gemi-, Empa-, or Gemi+Empa-treated groups. ^†^*p* < 0.05, ^††^*p* < 0.01, and ^†††^*p* < 0.001 indicate results with statistically significant differences between Empa- and Gemi+Empa-treated groups.

LPS induces the macrophage activation and the release of proinflammatory mediators, such as PGE_2_ and nitric oxide (NO), by upregulation of COX-2 and iNOS mRNA expression [37–39]. We confirmed that PGE_2_ production increased significantly in RAW 264.7 macrophages stimulated with LPS compared to cells without LPS induction. In contrast, empagliflozin and gemigliptin (individually and combined) inhibited PGE_2_ production in LPS-activated RAW 264.7 macrophages (Figure 2(a)). COX-2 mRNA and protein expression also increased markedly after LPS stimulation. Consistent with the decrease in PGE_2_ protein, the LPS-induced upregulation of COX-2 mRNA expression was attenuated after treatment with empagliflozin or gemigliptin (Figure 2(b)). mRNA expression of another important inflammatory enzyme, iNOS, was also downregulated by empagliflozin or gemigliptin (Figure 2(c)). Consequently, COX-2 and iNOS proteins were reduced in LPS-stimulated RAW 264.7 macrophages treated with empagliflozin or gemigliptin, indicating that the reduction of PGE_2_ protein was due to the suppression of the gene responsible for expression (Figures 2(d) and 2(e)).

### 3.3. Empagliflozin and Gemigliptin Reduced LPS-Induced Proinflammatory Cytokines and Chemokines in RAW 264.7 Macrophages

**Figure 3:**
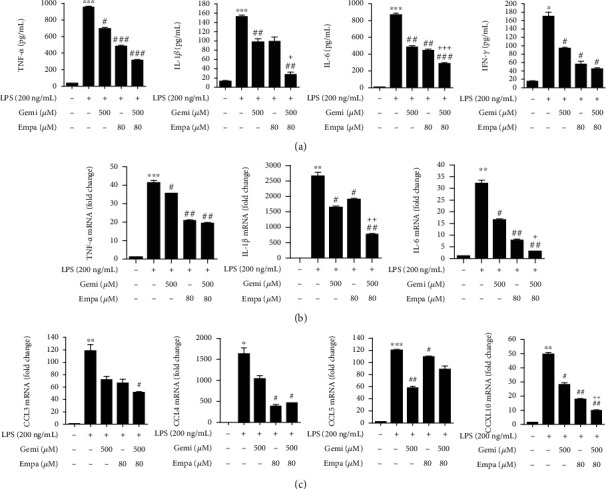
Empagliflozin and gemigliptin inhibited LPS-stimulated proinflammatory cytokines production and attenuated cytokines and chemokines mRNA expression in RAW 264.7 macrophages. (a) The amounts of TNF-*α*, IL-1*β*, IL-6, and IFN-*γ* in the conditioned medium were measured using ELISA after 48 h treatment. (b) TNF-*α*, IL-1*β*, IL-6, (c) CCL3, CCL4, CCL5, and CXCL10 mRNA expressions were analysed via real-time PCR after 4 h treatment. Data are presented as the means ± standard errors from three independent experiments. ^∗^*p* < 0.05, ^∗∗^*p* < 0.01, and ^∗∗∗^*p* < 0.001 indicate comparison with the untreated control. ^#^*p* < 0.05, ^##^*p* < 0.01, and ^###^*p* < 0.001 indicate results with statistically significant differences between the LPS-stimulated control and Gemi-, Empa-, or Gemi+Empa-treated groups. ^†^*p* < 0.05, ^††^*p* < 0.01, and ^†††^*p* < 0.001 indicate results with statistically significant differences between Empa- and Gemi+Empa-treated groups.

We confirmed increased production of inflammatory cytokines, such as TNF-*α*, IL-1*β*, IL-6, and IFN-*γ*, in the supernatant of LPS-treated RAW 264.7 macrophages [40]. These cytokines were reduced when the macrophages were treated with empagliflozin or gemigliptin, and an additional reduction was observed in empagliflozin plus gemigliptin-treated macrophages (Figure 3(a)). LPS-stimulated RAW 264.7 macrophages naturally upregulated the mRNA expression of proinflammatory cytokines (TNF-*α*, IL-1*β*, and IL-6) and chemokines (CCL3, CCL4, CCL5, and CXCL10). Treatment with empagliflozin or gemigliptin reversed the mRNA overexpression of these cytokines and chemokines in LPS-induced RAW 264.7 macrophages (Figures 3(b) and 3(c)). The effect of the empagliflozin plus gemigliptin combination was significantly greater than that of empagliflozin or gemigliptin alone.

### 3.4. Impact of Empagliflozin and Gemigliptin on NF-*κ*B, MAPK, and STAT Signalling Pathways in RAW 264.7 Macrophages

LPS activated various transcription factors associated with the inflammatory response in RAW 264.7 macrophages. Therefore, we conducted Western blot analysis of NF-*κ*B, MAPKs, and STAT signalling pathways to clarify the potential mechanisms of action of SGLT2 and DPP-4 inhibitors in the LPS-induced release of proinflammatory mediators, such as cytokines and chemokines [41–43]. We also used dexamethasone, a well-known anti-inflammatory reagent, as a positive control to compare the efficacy of empagliflozin and gemigliptin [44, 45].

**Figure 4:**
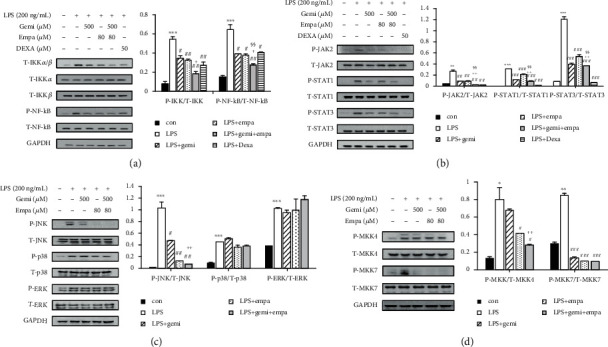
Empagliflozin and gemigliptin attenuated the NF-*κ*B, JNK, and STAT signalling pathways in RAW 264.7 macrophages. LPS-stimulated RAW 264.7 macrophages were treated with empagliflozin (Empa), gemigliptin (Gemi), empagliflozin plus gemigliptin (Empa+Gemi), or 50 *μ*M dexamethasone (DEXA) for 4 h. Here, DEXA served as the positive control for the anti-inflammatory reagent. (a) IKK*α*/*β*, NF-*κ*B, (b) JAK2, STAT1/3, (c) MAPKs, and (d) MKK4/7 signalling pathways in the cell lysates prepared were measured using Western blotting. Relative expression of each protein was compared. Data are presented as the means ± standard errors from three independent experiments. ^∗^*p* < 0.05, ^∗∗^*p* < 0.01, and ^∗∗∗^*p* < 0.001 indicate comparison with the untreated control. ^#^*p* < 0.05, ^##^*p* < 0.01, and ^###^*p* < 0.001 indicate results with statistically significant differences between the LPS-stimulated control and Gemi-, Empa-, or Gemi+Empa-treated groups. ^†^*p* < 0.05, ^††^*p* < 0.01, and ^†††^*p* < 0.001 indicate results with statistically significant differences between Empa- and Gemi+Empa-treated groups. ^§^*p* < 0.05, ^§§^*p* < 0.01, and ^§§§^*p* < 0.001 indicate results with statistically significant differences between DEXA- and Gemi+Empa-treated groups.

NF-*κ*B is a crucial transcription factor for proinflammatory mediators in macrophages [46]. NF-*κ*B phosphorylation was suppressed in RAW 264.7 macrophages treated with empagliflozin or gemigliptin. We also confirmed that empagliflozin and gemigliptin (individually and combined) inhibited IKK phosphorylation, which is a central regulator of NF-*κ*B activation, in LPS-activated RAW 264.7 macrophages (Figure 4(a)).

STATs are another important group of transcription factors in a variety of cytokines and growth factors for mediating pro- and anti-inflammatory responses [43]. STAT1 phosphorylation was attenuated in RAW 264.7 macrophages treated with empagliflozin or gemigliptin. However, gemigliptin was the more potent suppressor of STAT1/3 phosphorylation compared with empagliflozin (*p* < 0.05). Empagliflozin and gemigliptin (individually and combined) inhibited LPS-induced JAK2 phosphorylation in macrophages (Figure 4(b)).

MAPKs mediate biological processes and cellular responses to external stimuli, such as LPS [42, 47]. The JNK, p38, and ERK phosphorylation significantly increased in RAW 264.7 macrophages after LPS induction. Neither empagliflozin nor gemigliptin affected p38 and ERK phosphorylation; meanwhile, empagliflozin and gemigliptin (individually and combined) markedly attenuated JNK phosphorylation in RAW 264.7 macrophages activated by LPS. This effect of JNK phosphorylation was more pronounced in empagliflozin treatment (Figure 4(c)). To further determine the MAPK kinases involved in the JNK pathway inhibition, we examined MKK4 and MKK7, which cooperate in JNK activation. Empagliflozin significantly suppressed both MKK4 and MKK7 phosphorylation in RAW 264.7 macrophages stimulated by LPS, while gemigliptin mainly inhibited MKK7 activation (Figure 4(d)).

## 4. Discussion

**Figure 5:**
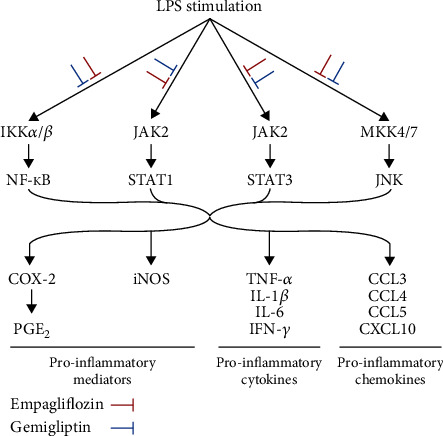
Proposal of a proinflammatory pathway inhibited by empagliflozin (SGLT2 inhibitor) and gemigliptin (DPP-4 inhibitor) in LPS-activated RAW 264.7 macrophages.

This study confirmed potent anti-inflammatory functions and concurrently demonstrated an anti-inflammatory pathway of SGLT2 and DPP-4 inhibition in LPS-activated RAW 264.7 macrophages. Empagliflozin and gemigliptin concurrently reduced proinflammatory cytokine and chemokine release and gene expression via the IKK/NF-*κ*B, JAK2-STAT1/3, and MKK4/7-JNK pathways in LPS-stimulated RAW 264.7 macrophages (Figure 5).

During inflammation, a host defense mechanism against various harmful stimuli, activated macrophages play an important role by producing several proinflammatory cytokines, such as TNF-*α*, IL-1*β*, IL-6, and IFN-*γ*, and inflammatory mediators, including NO and PGE_2_ [48]. However, chronic low-grade inflammation may result in metabolic syndrome, diabetes, cardiovascular disease, and cancer [49–52]. Currently, antidiabetic drugs are in the spotlight for their anti-inflammatory properties [53]. There is a meta-analysis to show C-reactive protein and proinflammatory cytokine IL-6 reduction in people treated with at least one SGLT2 or DPP-4 inhibitor among patients confirmed with coronavirus infection [54].

One SGLT2 inhibitor, canagliflozin, but neither dapagliflozin nor empagliflozin, inhibited proinflammatory cytokine IL-6 in an AMP-activated protein kinase- (AMPK) dependent manner in human endothelial cells (HUVECs) [55]. Canagliflozin also diminished proinflammatory cytokines, such as TNF-*α*, IL-1*β*, and IL-6, released by AMPK activation in LPS-treated RAW 264.7 macrophages [55]. In our study, empagliflozin decreased secretion of the proinflammatory mediators and downregulated the mRNA levels of COX-2, iNOS, TNF-*α*, IL-1*β*, and IL-6 in LPS-induced RAW 264.7 macrophages compared to LPS alone. Additionally, we confirmed its inhibitory effect on CCL3, CCL4, CCL5, and CXCL10 mRNA expression.

This divergence of empagliflozin according to the cell lines could be explained by the different distributions of SGLT1 and SGLT2, and the different concentrations used 1 *μ*M in HUVECs vs. 80 *μ*M in RAW 264.7 macrophages. Xu et al. [17] recently confirmed SGLT2 protein expression in RAW 264.7 macrophages, and in another study, the selectivity for SGLT2 of empagliflozin (half maximal inhibitory concentration, IC_50_ 3.1 nM) was similar to that of canagliflozin (IC_50_ 2.7 nM), but the selectivity for SGLT1 (IC_50_ 8,300 nM) was much weaker than that of canagliflozin (IC_50_ 710 nM) [56].

According to other study groups [57], canagliflzoin reduced proinflammatory cytokines by lowering hexose kinase II (HKII) and blocking ERK phosphorylation, but not affecting NF-*κ*B in LPS-stimulated human coronary artery endothelial cells (HCAECs). Dapagliflozin also suppressed iNOS, TNF-*α*, IL-1*β*, and IL-6 mRNA expression by attenuating the NF-*κ*B transcription factor in diet-induced atherosclerosis in rat aortic arteries [58]. In the mouse hepatic inflammation model [59], empagliflozin reduced the number of F4/80^+^ M1 macrophages and the mRNA expression of proinflammatory cytokines by attenuating NF-*κ*B and JNK phosphorylation.

We also confirmed that empagliflozin inhibited NF-*κ*B phosphorylation like other SGLT2 classes. Additionally, we investigated another inflammatory signalling STATs pathway to elucidate an anti-inflammatory mechanism. Empagliflozin inhibited STAT1 and STAT3 phosphorylation and also suppressed its upstream JAK2 activation. Subsequently, we verified that empagliflozin blocked IKK, which is upstream kinase of NF-*κ*B. However, it only suppressed JNK signalling without affecting p38 and ERK and naturally inhibited MKK4 and MKK7, which are upstream molecules of JNK. Empagliflozin inhibited the MKK4/JNK pathway more significantly than gemigliptin.

Consequentially, we revealed that empagliflozin markedly decreased the number of CD80^+^ M1 macrophages and the secretion of proinflammatory cytokines and chemokines by blocking the IKK-NF-*κ*B, MKK4/7-JNK, and JAK2-STAT1/3 pathways in macrophages.

Soluble DPP-4 (sDPP-4) activates immune response by upregulating CD86 (M1 macrophage marker) expression in antigen-presenting cells (APCs) via interleukin-1 receptor-related kinase 1 (IRAK-1) and NF-*κ*B signalling pathways [60, 61]. A DPP-4 inhibitor, anagliptin, attenuated TNF-*α*, IL-1*β*, and IL-6 by inhibiting NF-*κ*B phosphorylation, and JNK and p38/activator protein 1 (AP-1) activation in LPS-induced RAW 264.7 macrophages [62]. Other DPP-4 inhibitors, alogliptin and vildagliptin, also reduced proinflammatory cytokines by blocking NF-*κ*B through ERK1/2 and JNK signalling pathways in LPS-stimulated RAW 264.7 macrophages, respectively [25, 63]. Gemigliptin reduced proinflammatory cytokines by attenuating NF-*κ*B and JNK signalling via an Akt- and AMPK-dependent mechanism in LPS-induced human monocytic THP-1 cells [35].

In the current study, we confirmed the attenuation of proinflammatory cytokines (COX-2, iNOS, TNF-*α*, IL-1*β*, IL-6, and IFN-*γ*) and chemokines (CCL3, CCL4, CCL5, and CXCL10) by inhibiting NF-*κ*B and JNK phosphorylation in LPS-stimulated RAW 264.7 macrophages, but not affecting p38 and ERK phosphorylation. We demonstrated that gemigliptin inhibited NF-*κ*B phosphorylation through blocking IKK activation, known as a suppressor of NF-*κ*B. MKK4 and MKK7 are known to be upstream signalling components that are required for JNK, and we verified that inhibition of the JNK signal may result from blocking MKK7 signalling by gemigliptin. Notably, we newly proved that gemigliptin inhibits proinflammatory cytokine and chemokine production by reducing STAT1/3 phosphorylation, achieved by blocking the JAK2 signalling pathway, in LPS-induced RAW 264.7 macrophages.

These results suggest that gemigliptin improves inflammation in macrophages by inhibiting the activation of the IKK and NF-*κ*B, MKK7 and JNK, and JAK2 and STAT1/3 pathways. Gemigliptin was more potent in inhibiting the JAK2-STAT1/3 pathways than empagliflozin.

Only a few *in vivo* study reports on the anti-inflammatory effects of the combination of SGLT2 and DPP-4 inhibitors. Dual therapy, empagliflozin and linagliptin, synergistically ameliorated hepatic fibrosis and inflammation by reducing mRNA expression of proinflammatory cytokines in mouse liver [59]. Another combination therapy with dapagliflozin and saxagliptin ameliorated diabetic cardiomyopathy and diabetic nephropathy in mice by inhibiting proinflammatory cytokines and NLRP3/ASC inflammasome production [15, 64]. However, no studies have investigated whether the combination of SGLT2 and DPP-4 inhibitors directly affects the inflammatory response and its signalling pathways involved in macrophages. We clarified for the first time that the dual therapy, empagliflozin and gemigliptin, has stronger anti-inflammatory effects in RAW 264.7 macrophages.

## 5. Conclusions

In summary, we verified that empagliflozin and gemigliptin individually possess anti-inflammatory activity by reducing PGE_2_ and proinflammatory cytokine production, resulting from the inhibitory effects of COX-2, iNOS, cytokine, and chemokine mRNA expression in RAW 264.7 macrophages. These results were mediated by blocking the IKK and NF-*κ*B, MKK4/7 and JNK, and JAK2 and STAT1/3 signalling pathways. MKK4/JNK phosphorylation was prominently inhibited by empagliflozin, while JAK2-STAT1/3 activation was primarily suppressed by gemigliptin. All these anti-inflammatory effects were enhanced when empagliflozin was combined with gemigliptin compared to empagliflozin or gemigliptin treatment alone. This study is the first to show the direct effect of the anti-inflammatory mechanisms of empagliflozin and gemigliptin on macrophages and presents the stronger anti-inflammatory properties of combination therapy.

## Data Availability

The data used to support the findings of this study are available from the corresponding authors upon reasonable request.
